# Cardiomyocyte damage control in heart failure and the role of the sarcolemma

**DOI:** 10.1007/s10974-019-09539-5

**Published:** 2019-09-13

**Authors:** Ashraf Kitmitto, Florence Baudoin, Elizabeth J. Cartwright

**Affiliations:** Division of Cardiovascular Sciences, School of Medical Sciences, Faculty of Biology, Medicine and Health, Manchester Academic Health Science Centre, The University of Manchester, AV Hill, Dover Street, Manchester, M13 9PL UK

**Keywords:** Sarcolemma injury-repair mechanisms, Heart failure, T-tubules, Caveolae, Annexin, Dysferlin, MG53, EHD2

## Abstract

The cardiomyocyte plasma membrane, termed the sarcolemma, is fundamental for regulating a myriad of cellular processes. For example, the structural integrity of the cardiomyocyte sarcolemma is essential for mediating cardiac contraction by forming microdomains such as the t-tubular network, caveolae and the intercalated disc. Significantly, remodelling of these sarcolemma microdomains is a key feature in the development and progression of heart failure (HF). However, despite extensive characterisation of the associated molecular and ultrastructural events there is a lack of clarity surrounding the mechanisms driving adverse morphological rearrangements. The sarcolemma also provides protection, and is the cell’s first line of defence, against external stresses such as oxygen and nutrient deprivation, inflammation and oxidative stress with a loss of sarcolemma viability shown to be a key step in cell death via necrosis. Significantly, cumulative cell death is also a feature of HF, and is linked to disease progression and loss of cardiac function. Herein, we will review the link between structural and molecular remodelling of the sarcolemma associated with the progression of HF, specifically considering the evidence for: (i) Whether intrinsic, evolutionary conserved, *plasma membrane injury*-*repair mechanisms* are in operation in the heart, and (ii) if deficits in key ‘wound-healing’ proteins (annexins, dysferlin, EHD2 and MG53) may play a yet to be fully appreciated role in triggering sarcolemma microdomain remodelling and/or necrosis. Cardiomyocytes are terminally differentiated with very limited regenerative capability and therefore preserving cell viability and cardiac function is crucially important. This review presents a novel perspective on sarcolemma remodelling by considering whether targeting proteins that regulate sarcolemma injury-repair may hold promise for developing new strategies to attenuate HF progression.

## Introduction

Heart failure (HF) is a terminal illness for which there is no cure, with a heart transplant being the only option for survival. HF is a long-term, common complication, and end-point, of a range of cardiovascular diseases including myocardial infarction (MI) (Jhund and McMurray 2008) and diabetes (Cleland et al. 2005). HF mortality rates are poor, with approximately 50% of people dying within the first 5 years of diagnosis (Cleland et al. 2005), and with patients incurring recurrent and longer hospitalization (Minicucci et al. 2011). Significantly, the number of HF cases globally is on an upward trajectory due to an aging population and improved outcomes from coronary artery disease. Consequently HF is now considered to be an epidemic; with current estimates suggesting that worldwide more than 26 million people have HF (Ponikowski et al. 2014). While HF is a multi-faceted clinical syndrome, a common aetiology is the progressive development of morphological remodelling of the myocardium, which leads to a decline in contractile function and insufficient blood being pumped to meet the demands of the body. HF is also characterised by adverse structural alterations in response to pressure and volume overload; these include an increase in left ventricular (LV) mass, cardiomyocyte hypertrophy and inter-myocyte fibrosis, the extent of which can be correlated to systolic dysfunction and patient survival rates (Hill and Olson 2008). Replacement fibrosis is associated with cardiomyocyte death with extracellular matrix (ECM) accumulating in response to loss of cardiomyocytes which in turn contributes towards compromised cardiac function and contractility (Segura et al. 2014).

There is now a large body of data linking HF with cellular and molecular remodelling of the excitation–contraction (e–c) coupling apparatus (Zima et al. 2014). In brief, e–c coupling regulates Ca^2+^ influx into the cardiomyocyte, which stimulates Ca^2+^ release from intracellular stores (sarcoplasmic reticulum, SR) to activate contraction (systole) with re-uptake of Ca^2+^ into the SR and extrusion from the cell for relaxation (diastole). Underpinning e–c coupling and Ca^2+^ cycling is the precise spatial organisation of a host of ion channels, transporters, receptors and regulatory proteins, maintained in a specific geometry by specialised microdomains formed by the cardiomyocyte plasma membrane, termed the sarcolemma, and the SR. The sarcolemma and SR, the ‘sarcotubular’ network, provide the membrane framework to enable optimal geometric localisation of each Ca^2+^ cycling protein. While causal agents of HF are varied e.g. coronary artery disease, hypertension, metabolic disease, a primary pathology of the failing heart is impaired e–c coupling due to structural abnormalities of both the sarcolemma (Crossman et al. 2011) and SR (Pinali et al. 2013).

Therefore, here we shall focus upon the role of the sarcolemma in declining contractile performance and progressive cell death. Firstly, we shall examine if there is evidence of sarcolemma injury-repair mechanisms operating in the myocardium. Secondly, from a novel perspective, we will consider if deficits in the repair machinery occurr in HF and consequently may be a mediator of dysfunctional contractility and cell death due to loss of sarcolemma structural integrity.

## Sarcolemma remodelling and impaired contractility in HF

The unique cardiac sarcolemma morphology and protein and lipid composition allows the plasma membrane to act as a multi-functional organelle regulating a myriad of cellular processes including e–c coupling, as well as mediating the immune and inflammatory response through receptor signalling, substrate exchange and cytoskeletal anchoring. To co-ordinate these complex functions, and relevant to cardiac contraction, the sarcolemma is organized into several distinct structural microdomains; the transverse-tubules (t-tubules), caveolae and intercalated disc.

*T*-*tubules* are invaginations of the sarcolemma that penetrate the myocyte cell volume to propagate the action potential leading to the influx of calcium (Ca^2+^) to trigger calcium-induced-calcium release, underpinning synchronous e–c coupling. Multiple groups (Cannell et al. 2006; Heinzel et al. 2008; Lyon et al. 2009; Orchard et al. 2009), including our own (Pinali et al. 2013, 2017) have shown that loss and remodelling of t-tubules occurs in MI/HF and is a causative agent of hypo-contractility in HF patients (Hoydal et al. 2018; Louch et al. 2004). Moreover, it has been established that membrane remodelling is a *progressive* phenomenon with t-tubule disruption extending from the left to right ventricle as hypertrophy develops into HF (Wei et al. 2010). A consistent finding from studies of animal models and patients with cardiomyopathy and ischemic heart disease is that impaired e–c coupling is coincident with a loss of t-tubule density (Lyon et al. 2009).

*Caveolae* are small ‘Omega’ shaped incursions of the sarcolemma in regions enriched in cholesterol and sphingolipids. With diameters typically ranging from 50 to 200 nm these nanodomains provide a specialized environment for signalling proteins to cluster and orchestrate a host of cellular processes including vesicular transport, endocytosis, lipid homeostasis and mechanoprotection (Cheng and Nichols 2016). Abnormalities to caveolae morphology and protein content are associated with arrhythmogenesis and HF (Balijepalli and Kamp 2008); for an in-depth overview of caveolae and function in the heart see the recent article from Calaghan and colleagues (2018). A link between caveolae and t-tubules is also well established with caveolae proteins, in particular caveolin-3, shown to influence t-tubule structure and function. A recent study from Orchard and colleagues demonstrated how genetic ablation of caveolin-3 results in both structural deformation of the t-tubule network as well as a decreased I_Ca-L_ current, the initiator of e–c coupling (Bryant et al. 2018). Significantly, both hypertrophy and HF are associated with depressed levels of caveolin-3 (Feiner et al. 2011).

The *intercalated disc* is formed by the region of the sarcolemma between the poles of adjacent cells and is essential for maintaining the stability of the cardiac cell syncytium orchestrating the pumping action of the heart. The intercalated disc architecture is a complex structure composed of segments of folded membrane, termed plicae, separated by regions termed the nexus (Pinali et al. 2015). Each of these structural domains contain an array of proteins which form specialised complexes (desmosomes, adherens junctions and gap junctions) to provide inter-cardiomyocyte mechanical stability, transmission of electrical and metabolic signals and propagation of the action potential throughout the cardiomyocyte network [for a detailed review article of the structure and function of the intercalated disc in the heart see Bennett (2018)]. We have reported, as have other groups, that molecular and structural remodelling of the intercalated disc is a feature of HF (Pinali et al. 2015; Ortega et al. 2017). Deficits in expression of proteins localised to the intercalated disc have been implicated in a wide range of cardiovascular diseases including dilated cardiomyopathy and arrhythmogenic right ventricular cardiomyopathy (ARVC) (Zhao et al. 2019).

The brief summary above serves to illustrate that there is a large body of evidence to indicate that morphological rearrangements of sarcolemma microdomains are a pathological development of HF. However, the mechanisms and precipitating factors that promote sarcolemma remodelling remain to be fully elucidated.

## Sarcolemma integrity and necrosis in HF

A cumulative loss of cardiomyocytes has been demonstrated within the human failing heart with between a 10 and 100-fold percentage increase in cell death compared to hearts from healthy counterparts (Guerra et al. 1999; Saraste et al. 1999). Necrosis, a form of cell death is characterised by cell and organelle swelling, plasma membrane rupture and consequently *loss of sarcolemma integrity*, reduction in ATP and an influx of extracellular Ca^2+^. It should be noted that in addition to necrosis there are two other modes of cardiomyocyte death; apoptosis, and autophagy, pathways that have also been identified in animal models of HF and in patients. Apoptosis, unlike necrosis, is associated with cell shrinkage and reduced volume, and does not affect the integrity of the plasma membrane until the late stages of cell death. Similarly, autophagy does not lead to a change in cell size or stability of the sarcolemma, as it is a process that triggers lysosomal-mediated cannibalisation of a cell’s own building blocks, proteins and lipids, leading to the degradation of organelles e.g. the sarco-endoplasmic reticulum (S/ER) and Golgi. A detailed overview of cell death pathways in the heart is outside the scope of this review with many detailed articles available [e.g. see Moe and Marin-Garcia (2016)]. Additionally, since the focus of this review is the role of the sarcolemma we shall only consider the link between sarcolemma stability and necrosis.

Necrosis was initially believed to be an unregulated, passive process but is now known to be orchestrated by several signalling pathways; for detailed reviews see (Zhang et al. 2018; Zhu and Sun 2018). As reviewed by Zhu and Sun there are several types of regulated necrosis termed; ferroptosis, mitochondrial permeability transition (MPT)-dependent necrosis, necroptosis, NETosis, parthanatos and pyroptosis. Necroptosis and MPT-dependent necrosis have been implicated in the pathogenesis of MI, ischaemia–reperfusion (I/R) injury and HF. Following an MI, interventional strategies commonly include reperfusion to restore blood supply and rescue the damaged myocardium; however, I/R generates reactive oxygen species (ROS) (Hori and Nishida 2009) adding to the inflammatory response and to cell death (Eefting et al. 2004). Therefore, targeting the specific pathways involved in these processes has been suggested to have potential for leading to better outcomes (Dmitriev et al. 2013).

Investigations of patients with varying degrees of left ventricular, LV, systolic dysfunction show a progressive necrosis of cardiomyocytes during the transition from hypertrophy to HF (Hein et al. 2003). Receptor-interacting protein 1 kinase 3 (RIPK3) is a key molecule in the cell death pathway, forming a complex with RIPK1 and MLKL, and are proteins shown to be up-regulated in the failing human heart (Szobi et al. 2017). Inhibition of necroptosis through targeting RIPK3 results in improved cardiac function (Zhang et al. 2016) and thus is suggested to represent a novel treatment approach for MI, I/R injury and HF.

However, strategies to directly target the stability of the sarcolemma to counter necrosis are less well developed, although the concept of sarcolemma damage resulting from an MI is well established and exploited in clinical diagnosis, through monitoring the release of intracellular biomarkers (Garg et al. 2017). An MI leads to sarcolemma damage allowing Ca^2+^ influx, oxidation, depleted ATP and acidosis (Ishiharajima et al. 1986; Liu et al. 2015b). Studies using polyethylene glycol (PEG) copolymers, to enhance the sarcolemma integrity, have demonstrated that sarcolemma stability is linked to improved cardiac outcomes and reduction of cell death. For example, application of copolymers to neonatal rat ventricular myocytes challenged by I/R protocols led to a threefold reduction in cell death accompanied by preservation of β-adrenergic signalling (Malhotra et al. 2011). Subsequent studies (Bartos et al. 2016) reported how infusion of poloxamer 188 (P188) following occlusion of the coronary artery (porcine model of STEMI) coincident with commencement of reperfusion resulted in a reduction to infarct size, lower circulating Troponin I levels, less cell death and preserved mitochondrial function compared to controls. Although the molecular mechanisms of copolymer-sarcolemma interactions are not yet characterised it has been suggested that poloxamers provide protection against lipid peroxidation thereby enhancing sarcolemma stability, and function, whilst attenuating extracellular Ca^2+^ entry and overload.

However, there are many unanswered questions surrounding the mechanisms of membrane injury post-MI and in HF including (i) the size of wounds occurring in the sarcolemma due to ischaemic membrane injury; (ii) the underlying structural and molecular basis of sarcolemma injury and whether wounding of the sarcolemma is an impetus that triggers microdomain remodelling as an adaptive process; (iii) whether remodelling of sarcolemma structural microdomains precedes or predisposes cardiomyocytes towards necrosis and role in promoting necrosis.

## Sarcolemma injury and repair mechanisms

Since the plasma membrane is the first line of a cell’s defence against extracellular stresses (e.g. oxygen and nutrient deprivation, ROS, mechanical stress, inflammatory molecules, toxins) eukaryotic cells have evolved intrinsic membrane repair systems to reseal injury sites; for a detailed review see Cooper and McNeil (2015). Most of our current understanding of sarcolemma injury-repair mechanisms in striated muscle has come mainly from studies of skeletal muscle injury, through investigations of both small (< 100 nm) and large (> 100 nm and up to microns in size) wounding. Lipophilic holes which are small breaks in the lipid bilayer of a few nanometers or less can reseal spontaneously. However, in skeletal and cardiac muscle in which breaches are countered by an opposing mechanical force, through for example tethers to a cytoskeleton, a dedicated repair machinery is needed for healing the injury (Jimenez and Perez 2015). Studies of small injuries to skeletal muscle have revealed that the sealing mechanism involves the formation of two structural features at the wound site to mediate repair, the repair cap and the shoulder. These features are produced by the recruitment of several proteins including dysferlins, annexins, Eps 15 homology domain protein isoform 2, (EHD2) and mitsugumin 53 (MG53) to the injury site; together these proteins ‘plug the hole’, binding to the membrane in a process that is Ca^2+^-dependent, as reviewed by Cooper and McNeil (2015). Crucial to the ‘plugging’ of small membrane breaches is the presence, and exposure, of the membrane lipids phosphatidylserine and phosphatidylinositol 4,5-bisphosphate (PI(4,5)P_2_) (Demonbreun and McNally 2016). Other mechanisms which lead to small hole sealing in the plasma membrane include ‘shredding’ processes whereby the injury site is isolated through the formation of a bud, or bleb, in the damaged area of the plasma membrane, which then undergoes scission and is removed and released into the extracellular milieu. For a more extensive review of small hole repair see a recent article from Jimenez and Perez (2017).

It has been shown that eccentric contractions with high-force mechanical stress often lead to injury of the skeletal muscle plasma membrane with large wounds forming that are > 100 nm across, and which can extend to microns in size (McNeil and Khakee 1992). Under physiological conditions e.g. exercise-induced stress and when injury-repair mechanisms are intact the lesions that form in the plasma membrane lipid bilayer due to the increased mechanical force are sealed and function maintained (McNeil and Khakee 1992). Ca^2+^-induced exocytosis mechanisms are thought to be involved in the repair of these larger types of wounds resulting in ‘patching’ of the breach. One patching mechanism proposed to operate in skeletal muscle wound healing is caveolae proliferation, clustering, fusion and internalization driven, in part, by cleavage of sphingomyelin (an abundant lipid within the plasma membrane) (Andrews et al. 2014; Corrotte et al. 2013); we will discuss in the later sections of this review the evidence for a similar repair process in the heart and putative role of EHD2.

The section above serves to introduce the concepts of intrinsic sarcolemma wounding and repair processes, but it should be noted that the field of plasma membrane injury-repair is complex and extensive; with many excellent reviews of the topic available e.g. (Jimenez and Perez 2017). Indeed, there is now a large body research to confirm that under physiological conditions membrane repair processes in skeletal muscle act to counter injury to the sarcolemma, representing a form of cellular ‘rescue’ and pro-survival mechanism. However, what is less clear is how pathological conditions such as hypoxia, mechanical overload, oxidative stress, lead to changes to the physico-chemical properties of the striated muscle plasma membrane (i.e. lipid and protein content) altering the tensile properties of the bilayer, or how changes to expression levels, and/or post-translational modifications of the repair proteins are affected and contribute to cell death via necrotic pathways. While it has been demonstrated that the skeletal muscle t-tubules *are* susceptible to damage by eccentric stresses, resulting in adverse remodelling impairing transmission of the action potential and ultimately resulting in dyssnchronous contraction (Allen 2001; Edwards and Launikonis 2008), an association with defective membrane injury-repair mechanisms remains relatively unexplored. Interestingly, links between anti-oxidants and muscle health have been reported, with multiple animal models of vitamin E deficiency shown to exhibit muscle damage, impaired contractile function and increased levels of myocyte necrosis. For example, Howard et al. (2011), demonstrated an connection between oxidative stress and impaired injury-repair mechanism in skeletal muscle myocytes. Specifically, treatment with α-tocopherol (using concentrations in physiologically circulating ranges) was able to inhibit the effects of H_2_O_2_ and prevent repair-injury failure. Furthermore, myocytes that had been cultured in high glucose (30 mM) for 14 weeks (to mimic the effects of hyperglycemia) exhibited impaired resealing of holes generated by laser injury but when treated with α-tocopherol (200μM for 24 h) there was partial restoration of the injury-repair mechanisms.

Given that the heart is a continuously mechanically active organ there are few studies of plasma membrane injury-repair. However, McNeil and colleagues have demonstrated that under physiological conditions 25% of cardiac muscle cells exhibit disruptions to the sarcolemma and that injury to the sarcolemma results from the cyclic changes to pressure and volume with continuous variations in mechanical stress (Clarke et al. 1995). This study reinforced the link between mechanical stress and sarcolemma damage and showed that β-adrenergic stimulation, regulating the force and speed of contraction, led to a threefold increase in the frequency of wounding (Clarke et al. 1995). Given that cardiomyocytes are terminally differentiated, with a limited ability to regenerate, preservation of the sarcolemma integrity under pathological conditions is a crucial factor for preventing cell loss-of-function and necrosis. However, the mechanisms driving cardiac sarcolemma injury-repair processes under physiological conditions remain poorly defined, as is the impact upon wound healing processes and the consequences with a transition to pathophysiological stress.

Therefore, we will next consider; (i) the evidence for a putative link between sarcolemma remodelling in HF and impairment of intrinsic sarcolemma injury-repair mechanisms, and (ii) if a deficit in ‘wound-healing’ proteins may play a yet to be fully appreciated role in triggering necrosis.

## Role of sarcolemma ‘repair’ proteins in the heart

**Fig. 1:**
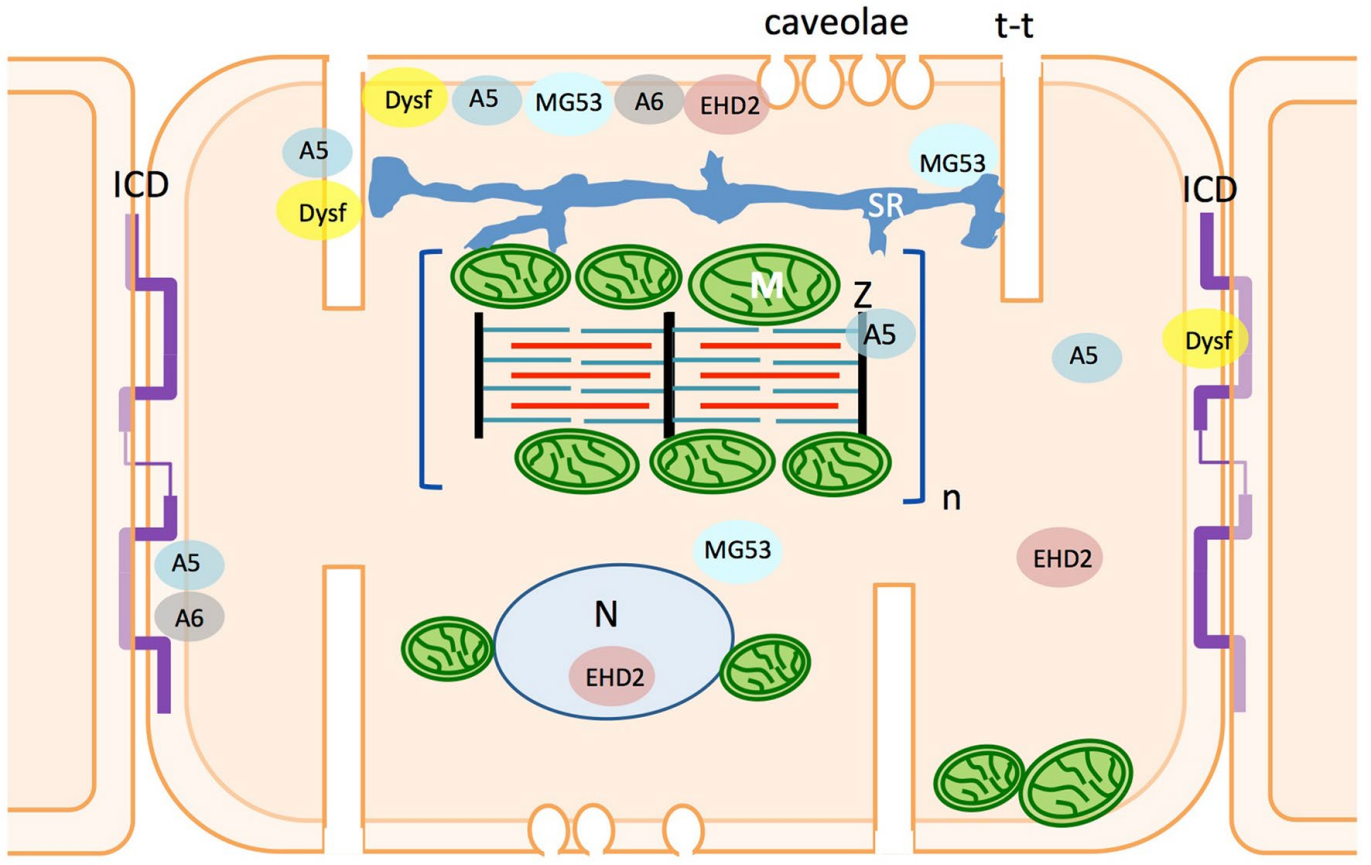
A representation of the putative spatial distribution of membrane-repair proteins within the cardiomyocyte. A cartoon of a cardiomyocyte illustrating microdomain organisation and organelle distribution with the sub-cellular localisation of annexins, dysferlin, EHD2 and MG53 populations indicated, as based upon the articles discussed in this review. (ICD = intercalated disc; M = mitochondria; N = nucleus; Z = Z-line)

This section will provide a summary of what is currently known about the function of key membrane repair proteins identified as important in skeletal muscle but within the context of the heart and HF. Specifically, we shall focus upon the roles of annexins, dysferlin, EHD2 and MG53. Figure 1 shows a cartoon of a cardiomyocyte and the putative spatial distribution of each of these repair proteins. As mentioned above the field of sarcolemma membrane-injury repair is vast (and outside the scope of this review article), therefore a summary of other protein machineries identified is provided in Table 1.

**Table 1:** Summary of plasma membrane repair proteins

Protein	Function within sarcolemma injury-repair processes	Examples of role in striated muscle repair
Annexins Most widely studied in this role; annexins 1, 2, 4, 5 and 6	Multifunctional roles; plugging of small wounds, recruitment of other repair proteins (dependent on injury size) and prevention of wound expansion	Numerous studies of skeletal muscle e.g. (Lennon et al. 2003) Limited data for cardiac muscle in this context.
Calpain I and II	Ca^2+^-activation of injury-repair proteins e.g. cleavage of dysferlin. Varying sensitivity of isoforms to intracellular [Ca^2+^] may be important for triggering different injury-repair machineries	Numerous studies of skeletal muscle e.g. (Mellgren et al. 2009) Studies of cardiac cell lines and in vivo cardiac specific ablation of Calpain I e.g. (Taneike et al. 2011)
Cytoskeletal machinery (i) Actin-myosin (ii) Microtubule motor proteins	(i) F-actin co-localises with EHD2 forming linker to caveolae; non-muscle myosin IIA mediates MG53 transport to injury site (ii) Kinesin motor activity (KIF5B) linked to spatio-temporal rates of vesicle fusion and recruitment of dysferlin Evidence for a complex co-operative process between actin-myosin-microtubules regulating and directing an actin-myosin organisation a ‘contractile ring’ to seal plasma membrane breaches	(i) Skeletal muscle (Marg et al. 2012); (Lin et al. 2012) (ii) Skeletal muscle e.g. (McDade et al. 2014)
Dysferlin	Plugging of ‘small’ holes (macromolecular complex formation with annexins, EHD2, MG53); putative role in exocytosis sealing of wounds sites through interaction with caveolin-3	Numerous studies of skeletal muscle e.g. (Bansal et al. 2003). Limited studies in cardiac muscle e.g. dysferlin-null mice (Han et al. 2007)
EHD2	Plugging of ‘small’ holes. Mediator of caveolae ‘patching for sealing of larger lesions	Numerous studies of skeletal muscle e.g. (Corrotte et al. 2013) Limited data for cardiac muscle/cardiomyocytes in this context.
ESCRT (i) Components of ESCRTIII (ALG-2, ALIX, Vsp4-ATPase) (ii) TRP cation channels (Mucolipin-1)	(i) Membrane sealing of small (< 100 nm) breaches (ATP dependent), mechanisms involve pore shredding. Although controversial, there is also evidence for involvement in repair of lesions up to 1 micron. (ii) Lysosomal exocytosis; regulates lysosomal Ca^2+^ release which mediates exocytosis and membrane repair. Binding partner of ALG-2	Limited studies (i) Skeletal muscle cell line (C2C12) e.g. (Scheffer et al. 2014) (ii) Mucolipin ^−/−^ mouse models develop muscle myopathy e.g. (Cheng et al. 2014)
MG53 (TRIM 72)	Plugging of ‘small’ holes. Complex interplay with caveolae proteins, caveolin-3, cavin-1, and polymerase I and transcript release factor (PTRF)	Numerous studies for both skeletal e.g.(Cai et al. 2009a) and cardiac muscle e.g. (Wang et al. 2010)
Transglutamilases	Putative involvement in stabilising annexins at repair sites	Data mainly for non- muscle cell types e.g. (Kawai et al. 2008). One in vitro study shows TG-2 cardioprotective in context of IPC (Vyas et al. 2018)

For a more detailed overview of each protein (and protein family) see reviews by Cooper and McNeil (2015), Jimenez and Perez (2017) and Andrews et al. (2014). Protein–lipid interactions and lipids such as phosphatidylserine, PI(4,5)P2, cholesterol and sphingomyelin also play a fundamental role in plasma membrane repair

### Annexins

The annexin family is composed of 12 isoforms, A1-11 and A13 with molecular mass ranging from ~ 35.5 to 76 kDa. The isoforms vary in length but each has a bipartite structure containing a conserved C-terminal domain referred to as the ‘core’ region and a variable N-terminal domain referred to as the ‘head’ region. Contained within the core are four, repeat, helical domains (with the exception of A6 which has a dual ‘core’) that form a disc-like structure; the convex side of the disc binds Ca^2+^ which mediates translocation to the membrane and phospholipid binding, positioning the concave region facing into the cytoplasm for facilitating annexin–protein interactions. Annexins, A1 and A2 through interactions with dysferlin, have been identified as one of the key molecular components for sealing small wound sites in skeletal muscle, by mediating vesicle fusion at the injury site in response to elevated intracellular Ca^2+^ (Lennon et al. 2003). A6 has also been shown to form part of the repair cap for orchestrating protein plug assembly in the injury-repair response in models of skeletal muscle myopathy (Swaggart et al. 2014); a process dependent upon both Ca^2+^ and actin binding (Demonbreun et al. 2016). A role for A5 in sealing wound sites in the skeletal muscle sarcolemma has also been demonstrated, in a process that is independent of other repair proteins such as dysferlin (Carmeille et al. 2016). It has been determined that A5 binds to exposed phosphatidylserine at the sarcolemma breach within the first few seconds of injury where it then self-assembles into crystalline-like 2-D arrays which in addition to ‘plugging’ the hole also serves to stabilise the injury site and prevent wound expansion (Bouter et al. 2011). Recently, A4 and A6 have been shown to work in concert to stabilise the edges of a wound (breaches spanning up to several microns in diameter) to generate tensile stress, by promoting curvature and constriction, to prevent enlargement of the lesion (Boye et al. 2017). Significantly, this seminal study demonstrated that to counteract expansion of the wound edges in response to the effects of A4 on membrane curvature A6 provides a balancing, stabilising, force, and is recruited several seconds ahead of A4 to the wound edges. Delineation of this mechanism involving A6 provided an explanation as to its unique structure containing two ‘cores’ showing that this architecture allows the protein to straddle the two membranes and thus play a key role in membrane repair. The above exemplify that the key roles of A1, A2, A4, A5 and A6 in skeletal muscle injury-repair are well characterised.

Luckcuck et al. (1997) using immunostaining methods revealed that A5 was located primarily around the nucleus in neonatal rat cardiomyocytes but in the adult myocardium had translocated to the sarcolemma and intercalated disc, a distribution that was independent of intracellular [Ca^2+^]. Further studies also identified populations of A6 and A5 within both atrial and ventricular cardiomyocytes, with A5 being more abundant in atria compared to the ventricles. However, A5 was distributed throughout the cell in contrast to A6 which was localised to the sarcolemma and intercalated disc (Doubell et al. 1993). Over 30 years ago an association between expression level changes to annexin A2, A5 and A6 (down-regulation of A6 and up-regulation of A2 and A5) was reported in patients with end-stage heart failure, although the functional and molecular significance of these changes were not clarified (Song et al. 1998). Further, A4, in addition A5 has been shown to be up-regulated in the failing human heart, with a relocation of A5 from the cytoplasm to the Z-line but with no change to levels or localisation of A6 (Matteo and Moravec 2000), in contrast with the earlier study from Song et al. Another study reported that in the healthy human heart A6 is the predominant isoform, compared to A2 and A5 but with increased expression of A2, A5 and A6 at the protein level in patients with idiopathic dilated cardiomyopathy (Benevolensky et al. 2000). In the non-failing hearts A2 was found predominantly in the interstitium and A5 and A6 at the sarcolemma, t-tubules and intercalated disc, but in contrast to other studies in the failing hearts A5 was virtually undetectable in the cardiomyocytes. Clearly, there are inconsistencies surrounding changes to expression levels and localisation of the annexins in the heart, in particular A6; these conflicting data may be due to the relatively small sample sizes employed due to difficulties in obtaining human tissue and also reflect different stages of the disease. While A7 is expressed in the myocardium (Magendzo et al. 1991), both its role and cellular location remains enigmatic; early data from non-muscle cells showed a role in exocytosis (Caohuy et al. 1996) and intriguingly, that it can assemble to form a Ca^2+^ channel (Pollard et al. 1991). However, an important role for A7 in the heart has been established with ablation of A7 linked to an increased susceptibility for developing atrial fibrillation, ventricular tachycardia and impaired Ca^2+^ homeostasis (Schrickel et al. 2007).

Clinically, the release of A5 into the circulatory system has been suggested as a valuable biomarker for cell death (Boersma et al. 2005). Employing an in vivo rat model of MI a correlation between myocyte apoptosis and A5 translocation to the sarcolemma and intercalated disc was reported with redistribution of A5 occurring as early as half an hour after occlusion of the LAD. Eight hours post-MI A5 was detected within the sarcolemma of border-zone cardiomyocytes and co-labelled with cells positive for apoptosis suggesting a key role for A5 in remodelling post-MI (Monceau et al. 2006). This report built upon previous in vitro studies showing A5 externalisation concomitant with increasing cell death when isolated rat cardiomyocytes were exposed to oxidative stress by treatment with H_2_O_2_ or staurosporine; blocking the effects of A5 extrusion to the outer surface of the sarcolemma reduced the expression of markers of apoptosis (Monceau et al. 2004). However, more recent studies have reported a therapeutic role for A5 in protecting against I/R damage (de Jong et al. 2018). Quax and colleagues (2018) showed, employing hypercholesterolemic ApoE*3-Leiden mice to mimic patients with high cholesterol/atherosclerosis, that administration of a daily dose of recombinant exogenous A5 immediately following MI-reperfusion (MI-R) surgery resulted in A5 accumulation within the infarct area. Importantly, the study showed that the infarct was reduced in size (27%) compared to untreated MI-R animals. Three weeks following MI-R the animals receiving A5 had an improved ejection fraction, a 28.5% increase compared to the untreated group, and with 42% less fibrosis. In terms of mechanisms it was proposed that the exogenous A5 binds to exposed phosphatidylserine in damaged cells and in doing so blocks the pro-death signalling cascade and also limits the inflammatory response as indicated by reduced macrophage infiltration, and levels of IL-6 in both the acute and late phases.

While, to-date, and to our knowledge, the role of annexins in cardiomyocyte membrane injury repair mechanisms has not been systematically dissected, there is clear evidence for links between changes to annexin isoform expression, cellular re-localisation and cell death in both HF patients and animal models of MI and MI-R. How molecular remodelling of annexins influences the development of cardiac pathologies in the context of sarcolemma injury-repair remains largely unexplored.

### Dysferlin

Dysferlin, a member of the ferlin family, is a transmembrane protein (with a molecular weight of ~ 230 kDa). Dysferlin is best characterised in the developmental role of muscle myopathies, specifically limb girdle muscular dystrophy (LGMD), Miyoshi myopathy (MM) and distal anterior myopathy as a result of gene (*DYSF*) mutations (Illa et al. 2001; Liu et al. 1998). LGMD and MM are generally characterised by muscle weakness and atrophy. A reduction in dysferlin expression and certain *DYSF* mutations in skeletal muscle are linked to aberrant sarcolemma repair mechanisms (Bansal et al. 2003). However, dysferlin does not form part of the multi-component dystrophin-glycoprotein complex important for maintaining the mechanical force between the sarcolemma, cytoskeleton and extracellular matrix. Duchenne muscular dystrophy, as a result of dystrophin deficiency, is characterised by an increase in shear and mechanical stress leading to sarcolemma instability and rupture as a result of eccentric and isometric contractions (Petrof et al. 1993). A report from Campbell and colleagues (2003) highlighted the critical role of dysferlin for membrane injury-repair mechanisms and the development of muscle myopathies but as a *separate* factor to compromised membrane fragility due to reduced expression of components of the dystrophin complex.

The C-terminal portion of dysferlin anchors it to the sarcolemma with interaction of dysferlin with proteins and lipids mediated by Ca^2+^ binding to the highly conserved C2 domains (a β-sandwich composed of 8 strands), of which there are seven. The C2 domains also facilitate protein–protein interactions which have been best characterised in skeletal muscle and include caveolin-3 (Matsuda et al. 2001), L-type voltage-gated calcium channel, A1 and A2 (Lennon et al. 2003), and MG53 (Cai et al. 2009c). Dysferlin has also been shown to have properties that can initiate tubule formation through association with phosphatidylinositol 4,5-bisphosphate (PI(4,5)P_2_) and thus is also proposed to play a crucial role in skeletal muscle t-tubule biogenesis (Hofhuis et al. 2017). Knockout mouse models of dysferlin exhibit a disordered sarcotubular network after injection of glycerol into the skeletal muscle to induce myopathy suggesting that dysferlin also contributes to skeletal muscle t-tubule stability (Demonbreun et al. 2014; Kerr et al. 2014). In summary, there are extensive studies establishing dysferlin as a crucial component of the skeletal muscle injury-repair machinery; for a detailed overview see Cooper and McNeil (2015).

In cardiac muscle, dysferlin is highly expressed and localised to the sarcolemma and intercalated disk (Chase et al. 2009) and the t-tubules forming a complex with caveolin-3 and the L-type voltage gated Ca^2+^ channel (Ampong et al. 2005). Transgenic mice deficient in dysferlin exhibited changes to the protein composition within the Z-disc, which would, in part, explain why the lusitropic response is compromised and why genetic mutations of dysferlin can also lead to the development of cardiomyopathies (Wenzel et al. 2007). A deficiency in dysferlin also results in the development of dilated cardiomyopathy (often a precursor to heart failure) exacerbated under conditions of mechanical stress induced by β-adrenergic stimulation (Han et al. 2007). Through monitoring the uptake of a membrane-impermeable fluorescent dye (FM1–43) into the cells (through the injury sites) and using live cell imaging to monitor resealing rates, Han and colleagues also demonstrated that control cardiomyocytes exhibit similar membrane repair processes in response to laser-induced focal wounding as skeletal muscle. Importantly, isolated cardiomyocytes from the dysferlin-null mice when similarly challenged were unable to reseal the lesions establishing a direct link between dysferlin and cardiomyocyte repair mechanisms. Interestingly, aging is associated with increased fibrosis and transgenic dysferlin knock-out mice exhibit progressive cardiac fibrosis (Chase et al. 2009; Han et al. 2007), which may imply a link between aging and impaired sarcolemma injury-repair mechanisms. Therefore, there is clear evidence that dysferlin has a role in regulating sarcolemma microdomain architecture and wound sealing, and that injury-repair mechanisms similar to those skeletal muscle are also operating in the heart.

### MG53

Since the first report describing a key role of MG53 for skeletal muscle plasma membrane repair (Cai et al. 2009a) there has been a host of other studies reinforcing these findings (Cai et al. 2009b; Weisleder et al. 2009). MG53 is a member of the tripartite motif family (TRIM) and so is often referred to as TRIM72. The architectural arrangement of MG53, comprising a RING finger, a B-box and coiled-coil region, allows it to also act as a regulator of ubiquitination pathways and as a ligase (Liu et al. 2015a). Ablation of MG53 in mice leads to age-linked progressive development of muscle myopathy and impaired sarcolemma injury-repair mechanisms (Cai et al. 2009a).

Compared to other membrane repair proteins identified in skeletal muscle MG53 is perhaps the best characterised in the heart. Wang et al. (2010) identified MG53 as a critical component for maintaining cardiomyocyte sarcolemma stability. They showed that genetic ablation of MG53 led to worse outcomes in response to I/R injury, with increased necrosis; a phenotype that was attributed to impaired sarcolemma resealing. Interestingly, the same study showed that *mg53*^−/−^ embryonic mice hearts did not exhibit abnormal cardiac function leading to the conclusion that the injury-repair mechanisms are activated only in response to adverse stimuli. Additionally, it was determined that cholesterol has a crucial role for recruiting cytosolic MG53 to the sarcolemma wound site. Other investigations of the mechanism of MG53 translocation to the sarcolemma determined that this process was independent of extracellular Ca^2+^ entry, or membrane stretching. Furthermore, movement of cytosolic MG53 to areas of disrupted sarcolemma was shown to be dependent upon redox sensing through MG53 Cys-242, a processes important for stabilisation of MG53 and subsequent activation of the repair process (Hwang et al. 2011). A study investigating the mechanisms of ischemic preconditioning (IPC) (Wang et al. 2010), an interventional procedure to minimise damage from I/R injury, provided further evidence of the cardioprotective role of MG53. Two distinct IPC signalling pathways have been defined; (1) the reperfusion injury salvage kinase (RISK) which is mediated via PI3K–Akt–GSK3β and ERK1/2 signalling (Hausenloy et al. 2005; Tong et al. 2000) and (2) the survivor activating factor enhancement (SAFE) pathway which is regulated via the action of tumor necrosis factor-α (TNF-α) and the JAK-STAT3 pathway (Lacerda et al. 2009). In-vitro I/R injury led to a loss in MG53 abundance accompanied with cell-death but introduction of IPC protocols rescued MG53 expression and showed an association with the RISK pathway through MG53 binding to caveolin-3 and PI3 K via the p85 subunit. Since these two seminal reports the cardioprotective role of MG53 in the heart has been reinforced in a study using adeno-associated virus (AAV) mediated overexpression of MG53 in a transgenic hamster (deficient in δ-sarcoglycan) with congestive HF. The group with elevated expression of MG53 exhibited improved heart function and had improved membrane repair capacity compared to the non-transfected group; as assessed by reduced infiltration of fluorescent dye indicating intact sarcolemma barrier function (He et al. 2012). The same study showed that MG53 overexpression was associated with an increase in dysferlin levels suggesting a possible co-operatively between the two proteins. The therapeutic potential of MG53 has been also demonstrated in a of porcine model of MI where intravenous delivery of recombinant MG53 via the jugular vein resulted in improved outcomes 4-weeks post-MI (Liu et al. 2015b). Treatment with recombinant MG53 either prior or post procedure led to lower Troponin I levels and smaller infarct sizes. It was proposed that recombinant MG53 targeted damaged sarcolemma by binding to phosphatidylserine clusters, which in response to sarcolemma damage are translocated to the extracellular leaflet of the membrane at injury sites. Interestingly, while MG53 was shown not to be involved in t-tubule development, Song and colleagues demonstrated that MG53 plays a crucial role in maintaining t-tubule organisation under conditions of pathological stress (Zhang et al. 2017). MG53 knock-out mice showed no loss of t-tubule structures and there was no impact upon the developmental time–course of the t-system network, confirmed by no change to cardiac function and Ca^2+^ handling properties. However, after 5-weeks exposure to LV pressure overload mice with genetic ablation of MG53 showed reduced survival rates, aberrant Ca^2+^ handling and extensive t-tubule remodelling when compared to wild-type controls. These data are consistent with the role of MG53 in maintaining the integrity of the sarcolemma and with it being a novel link between impaired membrane injury-repair mechanisms and t-tubule disruption. Very recently, MG53 was reported to be a regulator of NF-κB activity which is a transcriptional regulator of the K^+^ Channel Interacting Protein (KCHIP2) function, which may explain the sudden cardiac deaths reported in MG53 knockout mice following exercise training (Liu et al. 2019). However, despite these studies there still remains a lack of understanding of the mechanisms that regulate MG53 expression and down-regulation in response to pathological stress.

Whilst there is now a growing body of evidence in support of a cardioprotective role for MG53 there appears to be an exception in the context of diabetes. Cardiac MG53 is elevated in several animal models of diabetes but with overexpression shown to be a contributory factor to the development of the diabetic cardiomyopathy phenotype. MG53 by acting as an E3 ligase and through a direct interaction with the insulin receptor and insulin receptor substrate (IRS1) triggers receptor degradation leading to insulin resistance (Liu et al. 2015a). The study by Liu et al., highlighted a dual role for MG53 as an agent of membrane repair but also as a mediator of gene transcription, notably PPAR-α, which consequently contributes towards substrate inflexibility promoting FFA uptake, lipid accumulation, and ensuing cardiovascular complications. Thus, in the context of diabetic cardiomyopathy therapeutic strategies would be directed at attenuating the overexpression of MG53 and inhibition of the ligase activity.

In summary there are a multitude of studies providing evidence for a role of MG53 in t-tubule stability under conditions of stress, and that it is important for mediating cardiac sarcolemma injury-repair mechanisms in certain cardiac pathologies.

### EHD2

EHD2 is a member of a relatively newly identified family of four proteins (isoforms 1–4), and is a dynamin-related scaffold protein, localised to the plasma membrane in skeletal muscle and non-muscle cells (Naslavsky and Caplan 2011). All EHD isoforms have the same modular structure composed of an N-terminal region containing a GTPase domain, which is activated by ATP binding and not guanine nucleotides, separated from the C-terminal EH domain by a helical region (Daumke et al. 2007; Lee et al. 2005). The EH domain is considered the protein–protein interaction module which also contains a Ca^2+^ binding E–F hand motif. Crystal structures of soluble EHD2 show that it forms a dimer when ATP is bound (2QPT.pdb and 4CID.pdb) (Daumke et al. 2007). ATP binding to EHD2 is proposed to be a key step for the association of EHD2 dimers, followed by insertion into the membrane and assembly into oligomers (Daumke et al. 2007). Binding to PI(4,5)P2 has also been reported as necessary for EHD2 insertion into the plasma membrane (Simone et al. 2013). Oligomeric EHD2 is proposed to ‘bend’ membranes (Hoernke et al. 2017) but the size and structure of the oligomers remains to be resolved as does the basis of the mechanism resulting in membrane bending.

In addition to playing a key role in wound ‘clogging’ of small plasma membrane breaches (Cooper and McNeil 2015) numerous studies of non-muscle and skeletal muscle cells have established EHD2 as a regulatory switch to induce membrane curvature, regulate lipid homeostasis and limit caveolae motility (Moren et al. 2012; Shah et al. 2014; Simone et al. 2014; Stoeber et al. 2012). Further, these studies have led to the concept of a central role for EHD2 in ‘caveolae patching’ to seal large wounds (up to microns in diameter) in response to mechanical stress (Andrews et al. 2014; Corrotte et al. 2013). A detailed overview of caveolae clustering, fusion and wound sealing can be found in Andrews et al. (2014). In brief, a mechanism is proposed whereby a breach in the sarcolemma allows extracellular Ca^2+^ entry into the cell which triggers caveolae migration to the wound site where they assemble and merge together, a process that leads to constriction and closure of the lesion with endocytosis of the vesicles formed through caveolae fusion. A recent study applying mechanical stress to fibroblast cells (NIH 3T3) after EHD1, 2 and 4 silencing showed a loss of higher order caveolae clusters and an increased percentage of cells with plasma membrane rupture; thus establishing a clear link between caveolae clustering, mechanical stress and cell death (Yeow et al. 2017). What remains unclear is whether in response to injury there needs to be (i) in tandem de novo synthesis of caveolae to generate sufficient numbers of vesicles for sealing membrane wounds, or if whether existing caveolae are sufficient for sequestering to the breach site in the membrane, or (ii) how many caveolae are needed to reseal an injury or (iii) the size limit of the lesion that can be repaired using this mechanism.

Much less is known about EHD isoforms in the heart. An important study in 2010 by Mohler and colleagues first identified EHD isoforms (1–4) in all four chambers of the heart across species including man; describing a central role in membrane protein trafficking, with expression level changes linked to adverse membrane excitability in the infarcted heart (Gudmundsson et al. 2010). Since then further evidence has emerged for pivotal roles of EHD proteins in the heart with EHD3 expression reported to be differentially regulated in HF (Gudmundsson et al. 2012). We have reported that EHD2 localizes to sarcolemma and t-tubules and that expression levels in the LV are down regulated in the border-zone of a porcine model of MI one-month following the ischaemic event (Pinali et al. 2017). EHD1 knock-down in skeletal muscle leads to deformed t-tubules and is implicated as a scaffold for junctophilin-2 (JP2); a protein ‘bridge’ linking the t-tubule network to the junctional sarcoplasmic reticulum (Demonbreun et al. 2015). However, as yet no studies, similar to those of MG53 (Zhang et al. 2017), have been undertaken to investigate the role of EHD2 or EHD1 in skeletal or cardiac t-tubule development and organisation.

Caveolae proliferation in the heart is well established under conditions of ischemic preconditioning and is considered cardioprotective through amplification of pro-survival signalling pathways (Tsutsumi et al. 2008). Caveolae have also been reported in the heart to function as sarcolemma ‘reservoirs’ to buffer membrane tension (Sinha et al. 2011). A critical link between EHD2 and caveolae stability has also recently been demonstrated by Coetzee and colleagues showing EHD2 is necessary for stabilising caveolae, and for co-localisation of a sub-population of K_ATP_ channels leading to a proposed cardioprotective role of EHD2 against ischaemic damage (Yang et al. 2018). Other reports have characterised EHD2 in COS-7 cells as a transcriptional supressor, regulating gene expression via a nuclear localisation signal (NLS), a conserved nuclear export signal (NES) and SUMOylation site within the helical linker between the N-terminal and C-terminal domains (Pekar et al. 2012). Interestingly, while membrane insertion of EHD2 is ATP dependent Pekar et al. (2012) showed that nuclear entry of EHD2 is ATP independent. Furthermore, an association between mechanical stress, EHD2 mediated caveolae clustering and gene transcription has also been shown in HeLa cells. The application of a 10% repetitive mechanical stretch to HeLa cells led to translocation of ~ 10% of EHD2 from caveolae to the nucleus, whilst acute stress led to ~ 45% redistribution of EHD2 with disassembly of caveolae clusters; a process that is cyclic upon application and removal of the stress (Torrino et al. 2018). The same study also reported that EHD2 nuclear translocation by mechanical stress resulted in enrichment of genes linked to cell division, cell-cycle checkpoints and caveolae genes as well as regulating Krüppel-like factor 7 (KLF7). Therefore, although the role of EHD2 in cardiac sarcolemma injury-repair has yet to be established (as are the mechanisms of wound healing in general) there are clear links between EHD2 and caveolae dynamics. Perhaps a component of the cardioprotective properties of caveolae proliferation in hypertrophy and heart failure may be in wound patching, which is yet to be characterised.

## Conclusion

**Fig. 2:**
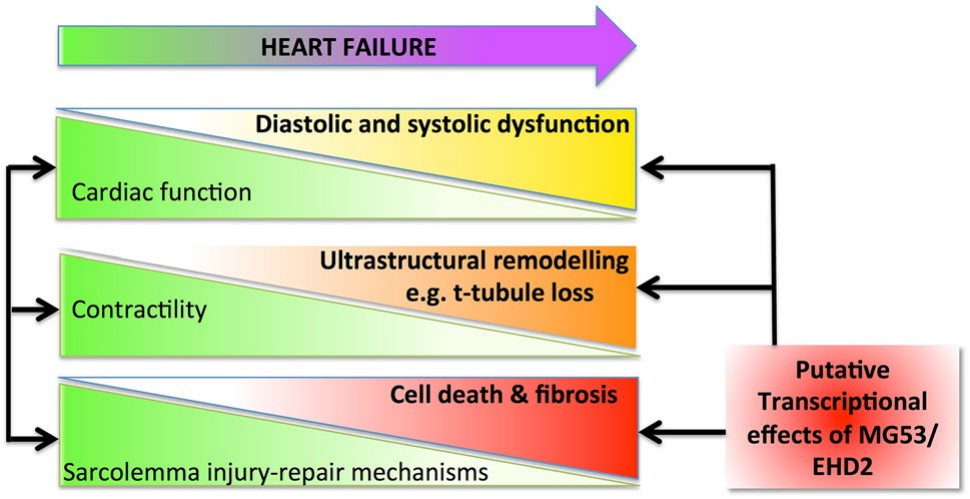
Schematic illustrating putative links between loss of cardiac function and heart failure progression. The figure highlights the interconnectivity between a decline in cardiac function and contractility and the development of diastolic and systolic dysfunction. A new tier to the pathology of HF is also illustrated; the concept that developing deficits in sarcolemma injury repair mechanisms, and proteins regulating these processes, may be a contributory factor

Here we have highlighted evidence indicating that sarcolemma injury-repair mechanisms are operating within the heart and that these are activated under conditions of cardiac stress including HF, MI, MI-R and I/R injury (see Fig. 2). While the injury-repair mechanisms remain to be fully delineated there is a wealth of data indicating that changes to expression levels of the putative ‘wound healing’ proteins are linked to the development of HF and complications of MI and I/R injury. Therefore, it is tempting to suggest that the molecular remodelling of these proteins in the setting of cardiovascular disorders may also be due a previously unappreciated link to the impaired ability of the cell to heal sarcolemma wounds. During the acute phase of an MI any intrinsic repair processes are likely to be out-stripped by the cascade of adverse signalling events, but targeting repair processes in chronic conditions may be beneficial for preventing cumulative cell death via necrosis. Additionally, although circumstantial, it is intriguing to note that the annexins, MG53, dysferlin and EHD1/2 all have links to sarcolemma microdomain (e.g. t-tubule and caveolae) stability and morphology and thus may also be involved in the adverse remodelling processes correlated to HF progression. An additional point of consideration is the recent discovery that the injury-repair machinery proteins MG53 and EHD2 are not only important for sarcolemma injury-repair, but may also have a secondary affect upon striated myocyte survival through mediating transcriptional signalling pathways. To summarise, we suggest that proteins implicated in intrinsic sarcolemma injury-repair may have potential therapeutic application for preserving plasma membrane integrity to prevent remodelling and possibly necrosis under conditions of chronic stress such as HF. However, a robust understanding of the factors that promote, and trigger membrane-healing pathways in cardiomyocytes is first necessary. In conclusion, this review has provided a novel outlook on potential driving factors of sarcolemma remodelling and necrosis.

## Abbreviations

AAV: Adeno-associated virus
ALG-2: Asparagine-linked glycosylation protein 2 homolog
ALIX: Programmed cell death 6-interacting protein
ApoE: Apolipoprotein E
ARVC: Arrhythmogenic right ventricular cardiomyopathy
ATP: Adenosine triphosphate
Ca^2+^: Calcium
*DYSF*: Dysferlin
e–c: Excitation–contraction
ECM: Extracellular matrix
EHD: Eps-homology domain protein
ERK1/2: Extracellular signal-regulated protein kinases 1 and 2
ESCRT: Endosomal sorting complex required for transport
FFA: Free fatty acid
GTP: Guanosine triphosphate
H_2_O_2_: Hydrogen peroxide
HF: Heart failure
I_Ca-L_: L-type calcium current
IL-6: Interleukin 6
IPC: Ischemic preconditioning
I/R: Ischaemia–reperfusion
IRS1: Insulin receptor substrate 1
JAK-STAT3: Janus kinases-signal transducer and activator of transcription 3
JP2: Junctophilin 2
K_ATP_: Channel ATP-sensitive potassium channel
KCHIP2: K^+^ channel interacting protein 2
KLF7: Krüppel-like factor 7
LAD: Left anterior descending artery
LGMD: Limb girdle muscular dystrophy
LV: Left ventricular
MG53: Mitsugumin 53
MI: Myocardial infarction
MI-R: Myocardial infarction-reperfusion
MLKL: Mixed-lineage kinase domain-like protein
MM: Miyoshi myopathy
MPT: Mitochondrial permeability transition
NES: Nuclear export signal
NF-κB: Nuclear factor kappa-light-chain-enhancer of activated *B* cells
NLS: Nuclear localisation signal
P188: Poloxamer 188
PEG: Polyethylene glycol
PI3K–Akt–GSK3β: Phosphatidylinositol 3–kinase–protein kinase B–glycogen synthase kinase 3 beta
PI(4,5)P_2_: Phosphatidylinositol 4,5-bisphosphate
PPAR-α: Peroxisome proliferator-activated receptor alpha
PTRF: Polymerase I and transcript release factor
RIPK1: Receptor interacting serine/threonine kinase 1
RIPK3: Receptor-interacting protein 1 kinase
RISK: Reperfusion injury salvage kinase
ROS: Reactive oxygen species
SAFE: Survivor activating factor enhancement
S/ER: Sarco-endoplasmic reticulum
STEMI: ST-elevation myocardial infarction
SUMO: Small ubiquitin-like modifier
TG-2: Tissue transglutaminase
TNF-α: Tumor necrosis factor-α
TRIM: Tripartite motif family
TRP: Transient receptor potential
T-tubules: Transverse-tubules
Vsp4: Vacuolar sorting protein 4
